# Differential Radiographic Response of Sagittal Foot Alignment to Early Weight Loss Following Sleeve Gastrectomy

**DOI:** 10.3390/medicina62050851

**Published:** 2026-04-30

**Authors:** Emre Erdoğan, Ömer Akay, Berk Koncalıoğlu, Mert Güler, Batuhan Gencer

**Affiliations:** 1 Department of General Surgery, Aktif International Hospital, 41275 Kocaeli, Türkiye; genelcerrahemreerdogan@gmail.com; 2 Department of General Surgery, Kocaeli City Hospital, 41000 Kocaeli, Türkiye; omer_akay_38@hotmail.com; 3 Department of Orthopaedics and Traumatology, Sultanbeyli State Hospital, 34935 Istanbul, Türkiye; berkkoncalioglu@gmail.com; 4 Department of General Surgery, Gaziosmanpaşa Training and Research Hospital, 34255 Istanbul, Türkiye; mertguler23@gmail.com; 5 Department of Orthopaedics and Traumatology, Marmara University Pendik Training and Research Hospital, 34899 Istanbul, Türkiye

**Keywords:** obesity, sagittal foot alignment, Meary’s angle, talar declination angle, calcaneal pitch angle, weight loss, medial longitudinal arch, body mass index

## Abstract

*Background and Objectives*: We aimed to evaluate early postoperative radiographic changes in sagittal foot alignment following laparoscopic sleeve gastrectomy and to investigate the association between early weight loss and sagittal foot alignment parameters. *Materials and Methods*: This study included 72 consecutive patients who underwent primary laparoscopic sleeve gastrectomy. Standardized lateral foot radiographs were obtained preoperatively and at the fourth postoperative month. Meary’s angle, calcaneal pitch, and talar declination angle were measured on all radiographs. Demographic and clinical variables, including age, sex, height, body weight, and body mass index (BMI), were recorded. *Results*: Meary’s angle demonstrated a significant postoperative decrease from 15° (IQR, 8°) to 11° (IQR, 12°) (*p* < 0.001), indicating improvement in medial longitudinal arch alignment. In contrast, no significant postoperative changes were observed in the calcaneal pitch (*p* = 0.227) or talar declination angles (*p* = 0.751). The proportion of patients within the normal range for all measured sagittal alignment parameters increased postoperatively, without showing statistical significance. Statistical analysis revealed that all postoperative sagittal alignment parameters showed significant correlation with preoperative values. Notably, postoperative Meary’s angle demonstrated a very strong positive correlation with preoperative Meary’s angle (r = 0.80, *p* < 0.001), whereas no significant correlation was identified between postoperative Meary’s angle and either postoperative weight or weight/BMI loss (*p* > 0.05). Although BMI loss showed a significant correlation with postoperative calcaneal pitch and talar declination angles, these correlations were weak to moderate (r = −0.403, and r = −0.362, respectively). *Conclusions*: Early postoperative body weight/BMI loss following sleeve gastrectomy is associated with modest, parameter-specific improvements in sagittal foot alignment, primarily reflected by changes in Meary’s angle, suggesting that the medial longitudinal arch may be more responsive to early postoperative unloading than other sagittal alignment parameters. The strong association between preoperative and postoperative measurements underscores the central role of baseline alignment in determining early postoperative outcomes.

## 1. Introduction

Obesity has emerged as a major global health concern, with well-established adverse effects on the musculoskeletal system, particularly in the lower extremities. Acquired foot deformities and foot pain are highly prevalent in obese individuals and constitute important determinants of reduced quality of life, given their direct impact on mobility, weight-bearing capacity, and activities of daily living. An increased body mass index (BMI) results in augmented mechanical loading across the foot, which may disrupt plantar pressure distribution, alter joint kinematics, and contribute to progressive changes in foot alignment [1]. These biomechanical alterations have been strongly associated with a reduction in medial longitudinal arch height and an increased predisposition to acquired flatfoot deformity [2,3]. Previous studies have demonstrated that elevated BMI is correlated with alterations in foot posture and a higher prevalence of flatfoot deformity, suggesting a direct relationship between obesity and structural changes in the foot [1,4,5].

Bariatric surgery, particularly sleeve gastrectomy, is widely recognized as one of the most effective interventions for achieving substantial and sustained weight loss. Beyond its metabolic benefits, postoperative weight loss has been associated with improvements in physical function and a reduction in musculoskeletal complaints; however, the magnitude and consistency of these effects remain controversial in the literature [6,7,8]. In 2023, Peres Pico et al. reported that weight loss following gastric bypass improved quality of life but was paradoxically associated with an increased prevalence of foot deformities [9]. On the contrary, Kim et al. demonstrated that, even in asymptomatic individuals, the talonavicular joint is closely associated with obesity and that increasing BMI exerts a detrimental effect on the medial longitudinal arch [10].

Furthermore, the early postoperative period following sleeve gastrectomy, characterized by rapid weight reduction, has not been sufficiently investigated with respect to structural foot alignment. Characterizing radiographic changes during this critical interval is of clinical importance, as such findings may reflect the earliest biomechanical adaptations of the foot and provide insight into the potential reversibility of obesity-related structural deformities [2,9,11]. Moreover, it remains unclear whether postoperative foot alignment is primarily influenced by the magnitude of weight loss or by baseline structural characteristics. Clarifying this issue is clinically relevant, particularly for informing obese patients during the postoperative period as to whether such deformities are predominantly structural in nature or potentially reversible following weight loss.

Accordingly, this study was designed to evaluate early postoperative radiographic changes in sagittal foot alignment following laparoscopic sleeve gastrectomy. Specifically, our primary objective was to assess changes in key alignment parameters and to investigate the association between early weight loss and sagittal foot alignment. Our secondary objective was to identify factors associated with postoperative alignment, with particular emphasis on the predictive role of preoperative alignment values. We hypothesized that weight loss would lead to a measurable improvement in sagittal foot alignment and that early postoperative weight loss would be associated with significant and favorable changes in sagittal foot balance.

## 2. Materials and Methods

Following the requisite approval from the Institutional Ethics Committee, this observational study included 72 consecutively enrolled patients who had undergone primary sleeve gastrectomy in 2025 and had been operated on by the same senior surgeon, and who were prospectively followed up. To ensure cohort homogeneity and minimize potential confounding factors, the study exclusively included patients who underwent primary laparoscopic sleeve gastrectomy. Patients who had undergone other bariatric procedures (e.g., Roux-en-Y gastric bypass or adjustable gastric banding), revision bariatric surgery, or combined surgical approaches were excluded from the study. Furthermore, patients with a prior history of foot or ankle surgery, as well as those with pre-existing structural foot deformities (including but not limited to rigid pes planus and pes equinovarus), were excluded to avoid bias in radiographic alignment measurements. All procedures involving human participants were conducted in accordance with the ethical standards of the institutional research committee and with the 1964 Declaration of Helsinki and its subsequent amendments. In this article, generative artificial intelligence (ChatGPT – GPT-5.3) was exclusively employed for rudimentary text editing tasks, such as spelling and punctuation correction. The utilization of large language models or other Generative AI products for the generation of text, data, or graphics, or to assist with study design, data collection, analysis, or interpretation, was not a part of the present study.

This study was designed within a prospective framework. In accordance with predefined inclusion and exclusion criteria, radiographs that were not part of routine clinical practice were obtained from all participants specifically for study purposes. However, as no randomization was performed and the data were not prospectively recorded in a dedicated study database, and since the data of patients who had been prospectively followed and regularly documented were retrospectively extracted from the hospital information system, the study was defined as a retrospective analysis of prospectively followed patients.

All sleeve gastrectomy procedures were performed laparoscopically by the same experienced bariatric surgeon (EE), who utilized standardized surgical techniques as previously outlined in the literature [12]. The perioperative management protocols, encompassing anaesthesia, antibiotic prophylaxis, and thromboprophylaxis, were implemented uniformly across all patients in accordance with institutional guidelines [12,13]. Postoperatively, patients were closely monitored, with particular attention paid to drain output, wound healing, and early complications. Following the implementation of stabilization procedures, patients were discharged from the care of a multidisciplinary team, which included a physiotherapist and a registered dietitian. The team implemented a standardized postoperative rehabilitation and nutritional support protocol.

The demographic and anthropometric characteristics of the patients included age, sex, height, preoperative body weight, and BMI. Early postoperative outcomes were assessed at the routine follow-up visit, which occurred approximately 4 months after surgery. At this stage, a re-evaluation of both body weight and BMI was conducted. The extent of weight loss was calculated and recorded for the purpose of subsequent correlation analyses.

For radiographic evaluation, patients were subjected to the same procedure of obtaining standardized lateral weight-bearing foot radiographs, both prior to surgery and at the early postoperative follow-up visit (median: 4 months). The assessment of sagittal plane foot alignment was conducted by means of three established radiographic parameters, measured in accordance with the definitions and techniques that had previously been published [14,15,16,17,18,19]. The lateral talus-first metatarsal angle (Meary’s Angle) was identified as the principal indicator of medial longitudinal arch alignment. This was measured as the angle between the longitudinal axes of talus and first metatarsi. The normal range of this parameter is defined as 0–4° [14,15]. The calcaneal inclination angle (calcaneal pitch), defined as the angle between the calcaneus and the supporting horizontal surface, has been identified as a significant factor in the evaluation of medial arch height. Its normal value, which is subject to variation due to factors such as race, is reported to range from 10° to 30° [14,15,16,17]. The talar declination angle, defined as the angle between the supporting surface and the longitudinal axis of the talus, has also been demonstrated to be a valuable metric in the assessment of longitudinal arch height. Its normal value is reported to be between 14° and 36° [14,18,19]. In order to enhance the reliability of measurement and reduce observer bias, all radiographic evaluations were conducted in conjunction with one junior and one senior orthopaedic surgeon who possesses extensive expertise in foot and ankle surgery. The evaluation process was conducted in accordance with the principles of consensus.

All statistical analyses were performed using IBM SPSS Statistics 26. The normality of data distribution was assessed using both visual (histograms and Q–Q plots) and analytical (Kolmogorov–Smirnov Test) methods. It was observed that all continuous variables exhibited a non-normal (skewed) distribution. The continuous variables were characterized using median, interquartile range (IQR), and minimum–maximum values, whilst the categorical variables were expressed as frequency (percentage). In order to facilitate inter-group comparisons, the Wilcoxon signed-rank test was utilized. Spearman’s rank correlation coefficient was utilized in the correlation analyses. A *p*-value of less than 0.05 was considered to be statistically significant. The strength of the correlation was interpreted as follows: 0.00–0.19 = very weak; 0.20–0.39 = weak; 0.40–0.59 = moderate; 0.60–0.79 = strong; 0.80–1.00 = very strong. The employment of negative Spearman correlation coefficients (r) was utilized to denote inverse relationships between variables.

## 3. Results

The median age of the study population was 48.5 years (IQR: 15). Of the 72 patients included in the study, 44 (61.1%) were female and 28 (38.9%) were male. The median preoperative body weight was 115.2 kg (IQR: 24 kg), and the median preoperative BMI was 44.1 kg per square meter (IQR: 4.0 kg/m^2^). At the postoperative fourth-month follow-up, the median body weight decreased to 90.45 kg (IQR: 16 kg) and the median BMI decreased to 31.8 kg/m2 (IQR: 5.1 kg/m^2^). A statistically significant reduction was observed in both body weight and BMI following laparoscopic sleeve gastrectomy. The median weight reduction was 27 kg (IQR: 12 kg), and the median decrease in BMI was 10 kg/m^2^ (IQR: 2.8 kg/m^2^) (*p* < 0.001 for both). A comprehensive overview of the patient’s demographic, anthropometric, and radiographic characteristics, both prior to and following the surgery, is provided in Table 1.

Regarding radiographic sagittal foot alignment, Meary’s angle demonstrated a statistically significant postoperative improvement. The median Meary’s angle exhibited a statistically significant decrease from 15° (IQR: 8°) preoperatively to 11° (IQR: 12°) postoperatively (*p* < 0.001). Conversely, no statistically significant differences were observed in the remaining sagittal alignment parameters. The median calcaneal pitch exhibited an increase from 26° (IQR: 10°) to 28° (IQR: 6°) (*p* = 0.227), while the median talar declination angle demonstrated a slight decrease from 15.5° (IQR: 5°) to 14° (IQR: 5°) (*p* = 0.751) (Table 1).

Although only Meary’s angle exhibited a statistically significant change in absolute angular measurements, an evaluation based on established normal reference ranges revealed a trend toward overall improvement in sagittal foot alignment across all parameters at the postoperative fourth month. Specifically, the proportion of patients with Meary’s angle within normal limits increased from 11.1% to 20.8%, the proportion of patients with normal calcaneal pitch increased from 55.6% to 66.7%, and the proportion of patients with normal talar declination angle increased from 66.7% to 72.2%. However, these enhancements did not attain statistical significance (*p* = 0.111, *p* = 0.171, and *p* = 0.469, respectively) (Table 2).

The implementation of correlation analysis revealed that postoperative radiographic alignment parameters exhibited a distinct correlation with preoperative alignment measurements. Postoperative Meary’s angle exhibited a very strong positive correlation with preoperative Meary’s angle (r = 0.80, *p* < 0.001), a very weak positive correlation with preoperative calcaneal pitch (r = 0.35, *p* = 0.003), and a strong negative correlation with preoperative talar declination angle (r = −0.67, *p* < 0.001). Conversely, postoperative Meary’s angle demonstrated no substantial correlation with either preoperative or postoperative body weight, BMI, or the extent of body weight/BMI change (*p* > 0.05 for all comparisons). Postoperative calcaneal pitch and talar declination angles exhibited statistically significant correlations with both preoperative radiographic alignment parameters and alterations in body weight and BMI. However, the observed correlations manifested as weak to moderate in strength (see Table 3 for details).

## 4. Discussion

Despite extensive research on the impact of excess body weight on sagittal foot alignment and flatfoot deformity, the effects of weight loss on sagittal alignment remain a subject of debate [6,7,8,9,10]. Furthermore, the extant literature on the subject is limited, with a paucity of studies investigating the early effects of weight loss and the reversibility of sagittal foot deformities. The present study’s primary strength and most significant contribution to the extant literature is its focus on the early postoperative period and its emphasis on the potential reversibility of sagittal foot alignment in obese patients. The primary finding of this study was that early weight loss following sleeve gastrectomy was associated with a significant improvement in Meary’s angle, whereas calcaneal pitch and talar declination angles remained unchanged. This indicates that the radiographic response of the foot to rapid postoperative unloading is selective rather than global. Conversely, while not reaching statistical significance, the rise in the number of patients whose measurements fell within normal ranges across all parameters is noteworthy and may signal a trend toward overall enhancement in sagittal alignment. A salient finding of this study is the observation that postoperative sagittal alignment parameters exhibited a strong correlation with their preoperative values. The substantial correlation between preoperative and postoperative measurements underscores the pivotal role of baseline alignment in determining early postoperative outcomes.

In the present study, it was observed that Meary’s angle exhibited a significant decrease postoperatively, a finding that is biomechanically plausible. Obesity has been associated with increased plantar loading, flatter foot posture, reduced inversion–eversion motion, and elevated plantar pressures, particularly in the midfoot and forefoot regions [1,20,21]. In this context, the postoperative decrease in Meary’s angle observed in our cohort likely reflects an early structural response of the medial longitudinal arch to reduced mechanical demand. The increase in the proportion of patients within the normal Meary’s angle range, from 11.1% preoperatively to 20.8% postoperatively, supports the interpretation that this change is not merely statistical but also anatomically meaningful [3,22]. Conversely, the calcaneal pitch and talar declination angles demonstrated no significant alterations. This dissociation is a pivotal finding of the present study. Meary’s angle directly reflects the alignment between the talus and the first metatarsal and thus may exhibit heightened sensitivity to short-term changes in load distribution. In comparison, calcaneal pitch and talar declination angles likely represent more stable hindfoot characteristics or osseous traits that may require a longer period to exhibit measurable adaptation. This interpretation is consistent with the extant literature on flatfoot, which suggests that individual radiographic parameters are indicative of distinct aspects of deformity rather than interchangeable measures of a single construct [3,23,24]. Conversely, while the numerical alterations and distributional shifts did not attain statistical significance, the increase in the number of patients within normal ranges across all parameters suggests that even early postoperative weight loss may contribute to an overall improvement in sagittal foot alignment. To more clearly define the effects of rapid early weight loss on sagittal foot alignment, larger-scale, prospective, and randomized studies with longer follow-up durations are needed.

Another key observation of this study was that preoperative radiographic alignment emerged as the strongest predictor of postoperative foot alignment. Postoperative Meary’s angle demonstrated a significant correlation with the preoperative Meary’s angle. However, no correlation was observed between Meary’s angle and either body weight or BMI. Moreover, other postoperative radiographic parameters exhibited a more substantial correlation with baseline alignment compared to postoperative body weight or BMI. This finding suggests that while weight reduction can indeed induce quantifiable enhancements, enhanced postoperative alignment cannot be solely attributed to a decrease in BMI. Postoperative morphology remains significantly influenced by the preexisting structural architecture. This distinction is clinically significant because it signifies that postoperative foot alignment is influenced not solely by weight reduction but also by long-standing structural adaptations that are present prior to surgery. It is also important to emphasize that the inverse correlation between postoperative Meary’s angle and postoperative talar declination angle is also biomechanically consistent. As Meary’s angle underwent refinement, talar declination exhibited a tendency to diminish. Nevertheless, given that talar declination did not demonstrate a substantial alteration at the group level, it is plausible that this association is indicative of interindividual variability in alignment patterns, as opposed to a uniform postoperative correction across all sagittal parameters.

The present findings are largely consistent with extant bariatric literature, which demonstrates that weight loss leads to improvements in musculoskeletal pain, gait, mobility, and quality of life [9,25,26]. Baropodometric studies have demonstrated a reduction in plantar pressure and foot loading following postoperative weight loss [2,9,11,27]. The present study extends these findings by demonstrating that the effects of bariatric surgery may also be detectable at the radiographic level, particularly in Meary’s angle. Concurrently, the findings of this study indicate that structural adaptation is selective rather than generalized, a phenomenon that may elucidate the occurrence of functional recovery despite the absence of measurable changes in specific radiographic parameters [28].

It is also crucial to note that the absence of substantial alterations in calcaneal pitch and talar declination may be attributed to the comparatively brief duration of the follow-up period. Postoperative assessment was performed at a median of four months, which is likely sufficient to detect early unloading effects but may be insufficient to capture more extensive osseous or ligamentous remodeling. Extended follow-up periods may elucidate whether these parameters remain stable or undergo delayed adaptation [29,30].

It is crucial to avoid overinterpretation of the study findings. Although the findings of this study suggest that early weight loss leads to significant changes in Meary’s angle and that preoperative foot alignment is the strongest predictor of postoperative alignment, the lack of clinical outcomes and the exclusive reliance on radiographic parameters limit the clinical relevance of our results. Although the study may contribute meaningfully to existing literature, its implications remain constrained, and the concordance between radiographic findings and clinical outcomes should be investigated in future large-scale studies.

The present study is not without its limitations. Firstly, although all patients were prospectively followed, the retrospective design limits control over potential confounding factors. As mentioned before, the duration of the follow-up period (approximately 4 months) was comparatively brief, enabling the assessment of only the initial postoperative alterations. On the other hand, this was consistent with our aim of investigating the early effects of weight loss. Another important limitation is that the exclusion of patients with pre-existing foot deformities may limit the generalizability of the findings, particularly given the high prevalence of such conditions in obese populations. It is also important to emphasize that the study did not incorporate several key elements, including functional outcomes, plantar pressure measurements, gait analysis, and patient-reported outcome measures (PROMs). While the use of PROMs has the potential to offer significant insights into the clinical relevance of weight loss and sagittal alignment changes, they were not incorporated due to the high prevalence of musculoskeletal pain in obese patients. This condition has the potential to introduce substantial confounding, thereby limiting interpretability. In addition, multiple correlation analyses were performed without adjustment for multiple comparisons, which may increase the risk of type I error. Finally, the decision to utilize solely three sagittal alignment parameters constitutes a substantial limitation. Conversely, a thorough review of the extant literature reveals that more than 20 radiographic parameters have been described for the purpose of assessing sagittal foot alignment. However, it was not feasible to evaluate all these parameters simultaneously. Future multi-center studies with larger cohorts and longer follow-ups, incorporating a broader range of sagittal parameters, may provide a more comprehensive understanding of the effects of obesity and weight loss on foot alignment.

## 5. Conclusions

In summary, early weight reduction after sleeve gastrectomy is associated with a substantial enhancement in Meary’s angle. Conversely, calcaneal pitch and talar declination angles exhibited no statistically significant alterations, suggesting that Meary’s angle responds more rapidly to early postoperative unloading compared to other sagittal alignment parameters. It is imperative to note that preoperative radiographic alignment was identified as the most robust predictor of postoperative alignment, suggesting that postoperative foot morphology is influenced by a combination of weight reduction and baseline structural characteristics.

## Figures and Tables

**Table 1:** Demographic, anthropometric, and radiographic characteristics of the patients.

Parameter		Median (Interquartile Range)	Range (Minimum–Maximum)
**Age** (years)		48.5 (15)	18–59
**Gender** *	**Female**	44 (61.1%)	
	**Male**	28 (38.9%)	
**Patient Height** (cm)		165 (18)	150–180
**Patient Weight** (kg)	Preoperative	115.2 (24)	94–152
	Postoperative	90.45 (16)	72–119
	*p*-value **	***<0.001***	
**Weight Change** (kg)		27 (12)	18–38
**Body Mass Index** (kg/m^2^)	Preoperative	44.1 (4.9)	35.3–50.1
	Postoperative	31.8 (5.1)	27.8–38.5
	*p*-value **	***<0.001***	
**Body Mass Index Change** (kg/m^2^)		10 (2.8)	7.1–12.8
**Meary’s Angle** (°)	Preoperative	15 (8)	1–37
	Postoperative	11 (12)	4–22
	*p*-value **	***<0.001***	
**Calcaneal Pitch** (°)	Preoperative	26 (10)	9–41
	Postoperative	28 (6)	10–40
	*p*-value **	0.227	
**Talar Declination Angle** (°)	Preoperative	15.5 (5)	6–22
	Postoperative	14 (5)	7–23
	*p*-value **	0.751	

* Categorical variables were presented as frequency (percentile). ** Statistical significance *p* value between preoperative and postoperative measurements.

**Table 2:** Comparison of changes in sagittal foot alignment based on normal ranges.

		Preoperative	Postoperative	*p*
**Meary’s Angle** (NR: 0–4°)	Within Range	8 (11.1%)	15 (20.8%)	0.111
	Out of Range	64 (88.9%)	57 (79.2%)	
**Calcaneal Pitch** (NR: 10–30°)	Within Range	40 (55.6%)	48 (66.7%)	0.171
	Out of Range	32 (44.4%)	24 (33.3%)	
**Talar Declination Angle** (NR:14–36°)	Within Range	48 (66.7%)	52 (72.2%)	0.469
	Out of Range	24 (33.3%)	20 (27.8%)	

N: number of patients; NR: normal range; *p*: statistical significance value.

**Table 3:** Correlation between preoperative-postoperative sagittal foot alignment parameters and body weight/body mass index changes.

	Postoperative Meary’s Angle	Postoperative Calcaneal Pitch	Postoperative Talar Declination Angle
**Patient Height**	0.420	0.491	0.202
**Preoperative Weight**	0.772	0.110	0.619
**Preoperative Body Mass Index**	0.426	***0.014***	0.240
		*r = −0.29*	
**Postoperative Weight**	0.169	0.945	0.331
**Postoperative Body Mass Index**	0.153	***0.011***	0.798
		*r = −0.30*	
**Weight Change**	0.448	***0.004***	0.139
		*r = −0.34*	
**Body Mass Index Change**	0.094	***<0.001***	***0.002***
		*r = −0.40*	*r = −0.36*
**Preoperative Meary’s Angle**	***<0.001***	***<0.001***	***<0.001***
	*r = 0.80*	*r = 0.58*	*r = −0.46*
**Preoperative Calcaneal Pitch**	***0.003***	***0.002***	0.869
	*r = 0.35*	*r = 0.35*	
**Preoperative Talar Declination Angle**	***<0.001***	***<0.001***	***<0.001***
	*r = −0.67*	*r = −0.41*	*r = 0.42*
**Postoperative Meary’s Angle**	N/A	***<0.001***	***<0.001***
		*r = 0.44*	*r = −0.68*
**Postoperative Calcaneal Pitch**	***<0.001***	N/A	0.680
	*r = 0.44*		
**Postoperative Talar Declination Angle**	***<0.001***	0.680	N/A
	*r = −0.68*		

N/A: non-applicable. The values in the cells represent *p*-values indicating statistical significance. The Spearman correlation coefficient is shown for cells where the *p*-value is less than 0.05.

## Data Availability

The datasets generated and/or analyzed during the current study are stored in a private repository and are not publicly available; however, they may be obtained from the corresponding author upon reasonable request.

## Abbreviations

The following abbreviations are used in this manuscript:

BMI	Body mass index
GenAI	generative artificial intelligence
IQR	Interquartile range
PROM	Patient-reported outcome measurement
